# Symptoms of osteoarthritis influence mental and physical health differently before and after joint replacement surgery: A prospective study

**DOI:** 10.1371/journal.pone.0217912

**Published:** 2019-06-06

**Authors:** Thomas V. Perneger, Didier Hannouche, Hermès H. Miozzari, Anne Lübbeke

**Affiliations:** 1 Division of Clinical Epidemiology, Geneva University Hospitals, Geneva, Switzerland; 2 Division of Orthopedic Surgery and Traumatology, Geneva University Hospitals, Geneva, Switzerland; Monash University, AUSTRALIA

## Abstract

**Background:**

Patient-reported outcomes are increasingly used in evaluations of joint replacement surgery, but it is unclear if symptoms of osteoarthritis (i.e., pain and dysfunction) influence health perceptions similarly before and after surgery.

**Methods:**

In this prospective study based on a hospital-based arthroplasty registry, patients with primary total hip or knee arthroplasty (THA, N = 990, and TKA, N = 907) completed the WOMAC Pain and Function scales, and the SF12 Physical and Mental Component Scores (PCS and MCS), before surgery and one year later. Associations between WOMAC and SF12 scales were examined using mixed linear regression models.

**Results:**

All patient-reported outcomes improved following total joint arthroplasty, but the associations between symptom scales and global health perceptions were altered. Mental health scores at a given level of pain or function were lower after surgery than before, by about 4–5 points, a clinically meaningful and statistically significant difference. In contrast, the associations between WOMAC scales and the PCS remained stable. These findings were observed in both cohorts of patients.

**Conclusions:**

After total joint arthroplasty, mental health scores were lower than would have been expected given the symptomatic improvement. This suggests that relationships between patient-reported outcomes are context-dependent, and that care should be exerted when interpreting changes in patient-reported outcomes over time.

## Introduction

Patient-reported outcomes (PROs) such as pain and functional ability, perceptions of physical and mental health, and quality of life in a global sense, are increasingly used to assess the impact of joint replacement surgery [1–4]. PROs can be employed to guide clinical care decisions, monitor quality of care, perform between-hospital comparisons [5], or adjust reimbursement policies [6].

PROs are often understood to be linked causally; e.g., specific symptoms such as pain or impairment determine global perceptions of health, which in turn contribute to perceived quality of life [7]. If the relationships between symptoms and more global PROs were stable, it would be easy to predict the impact of symptom relief, such as can be afforded by joint replacement surgery, on general health. However, relationships between PROs may vary with context. E.g., the same level of impairment may be perceived differently if the problem is temporary rather than permanent, if the patient develops other health issues, or if the patient reevaluates his or her priorities [8–10]. In patients with degenerative joint disease, a key contextual variable is the occurrence of joint replacement surgery. Before surgery, some patients may consider osteoarthritis symptoms as temporary and curable, and thus more tolerable. It is currently unclear if the impact of pain or functional impairment on perceived health remains stable or changes after joint replacement surgery. This issue is important for the interpretation of changes in PROs following surgery.

In this study, we examined the stability of the relationship between patients’ symptoms (pain and functional impairment) and global perceptions of physical and mental health, by comparing the periods before and after hip or knee replacement surgery. We do not focus on the absolute changes in PROs brought on by surgery, but rather on the impact of surgery on the associations between PROs.

## Patients and methods

### Study design and sample

This prospective study used data collected by the Geneva Arthroplasty Registry [11]. The registry is based at the largest University hospital in Switzerland (Geneva University Hospitals), the only public hospital of the canton (state) of Geneva, which serves a population of about 500,000 inhabitants. The registry includes all patients treated with total arthroplasty of the hip or knee since 1996 and 1998, respectively. The registry team consists of a physician-epidemiologist, senior and junior orthopaedic surgeons, a data manager and information technology specialist, and medical secretaries. Data are entered on a daily basis retrieved from the same data sources (patient questionnaires, preoperative report from surgeon and from anesthesiologist, detailed operative report, discharge summary, standardized clinical follow-up forms). Main complications are double-checked by the data manager in charge of the hip registry. The numbers of arthroplasties performed and any main complication related to surgery are periodically verified by comparing the hospital diagnosis coding system with the registry data.

For this study we included all patients who underwent primary elective total hip arthroplasty (THA) or total knee arthroplasty (TKA) between 2010 and 2016, and who had a health status and functional outcome measurement performed pre-operatively and one year after surgery. We included one or two primary THAs or TKAs in a given patient, but excluded revision procedures.

### Study variables

The main dependent variables were mental and physical health status, measured by the Short Form 12-item (SF12) questionnaire [12], adapted from the French translation of the SF-36 [13]. This instrument yields two summary scores: a Mental Component Score (MCS), and a Physical Component Score (PCS). All 12 items contribute to each score, but are weighed differently. Item response weights were those of the original algorithm [12].Both scores have a mean of 50 in the general US population, and a standard deviation of 10; higher scores imply better health.

The main independent variables were the timing of the measurement (5–14 days before surgery, after 1 year) and the patients’ pain and function, measured by the Western Ontario and McMaster Universities Osteoarthritis Index (WOMAC) 12-item questionnaire [14, 15]. This instrument yields a Pain score, and a Function score, both between 0 (worst) and 100 (best).

Descriptive variables included patient sex and age (also categorized in 4 strata), the American Society of Anesthesiologists (ASA) score [16] (a physical status classification system that reflects the presence of no (1), mild (2), moderate (3) or severe (4) systemic disease), body mass index, current smoking status, medical comorbid conditions (heart disease, high blood pressure, diabetes mellitus), a numerical scale for pain in the affected joint (between 0, no pain, and 10, worst imaginable pain), and the University of California, Los Angeles (UCLA) numerical scale for activity level (between 1, wholly inactive and dependent on others, and 10, regularly participating in impact sports) [17].

The SF12, WOMAC, numerical scale for pain, and UCLA Activity scale were self-reported by the patient using a mailed questionnaire. The ASA score, weight, height, and smoking status were abstracted from the anesthesiologist’s pre-operative assessment (whether height and weight are actual measures or patient reported is not recorded). Patient age, sex and comorbidities were abstracted from the patient’s medical file.

### Statistical analysis

We included all eligible patients from the Registry, without an a priori sample size determination. We compared study participants to those who were excluded due to incomplete data, and compared the characteristics of patients who underwent primary THA and TKA. We reported the means and standard deviations (SD) of the SF12 and WOMAC scores at both points in time. We kept separate the analyses of THA and TKA patients, in order to verify the generalizability of the findings.

To describe associations between each WOMAC score and each SF12 score, we obtained scatterplots for each pair of variables, overlaying baseline and follow-up measurements. To represent the associations without imposing a pre-conceived shape we obtained non-parametric regression functions (Locally weighted regression and smoothing scatterplots) [18]. As visual inspection of these functions suggested that a simple linear model was reasonable, we obtained intercepts and slopes for linear regression models of each SF12 score on each WOMAC score, at both points in time. To facilitate the interpretation of the intercepts and slopes, the WOMAC scores were centered at 50 points and divided by 10: the intercept represents the expected SF12 score for a patient with a WOMAC score of 50, and the slope represents the gain in SF12 score for a difference of 10 points on the WOMAC score. The variance in each SF12 score explained by each WOMAC score was also reported (adjusted R^2^ from the linear model).

We used mixed linear models to estimate the change in intercept and slope between baseline (Time = 0) and follow-up (Time = 1). To account for the lack of independence of repeated observations, we included a random factor u_j_ for each patient j, and also a random factor v_k(j)_ for each joint k in patients who had successive operations of both joints (knees or hips). The “joint” random factor v_k(j)_ was nested within the “patient” random factor u_j_. Both random factors were used to model the intercept only, assuming that each patient (and each joint) has a given tendency to yield higher or lower SF12 scores, and that these individual tendencies are normally distributed with a mean of 0 and a variance estimated from the data. To facilitate the interpretation of the intercepts, we have centered the WOMAC scales at 50, and to magnify the slope coefficients we divided the centered scores by 10. As a result, the fixed intercepts correspond to patients with a WOMAC score of 50 (instead of 0), and the slope coefficients correspond to a difference in SF12 scores for an increment of 10 points on the WOMAC scale. E.g., for the MCS and the WOMAC Pain score, we used the following model:

$$MCS=b_{0}+u_{j}+v_{k(j)}+b_{1}∙Time+b_{2}∙Pain+b_{3}∙Time∙Pain+error$$

The fixed coefficient b_0_ is the mean intercept, u_j_ and v_k(j)_ are the individual and side-specific random effects, both centered at 0, and fixed coefficients b_1_, b_2_, and b_3_ represemt the effects of scientific interest. Thus b_0_ is the mean preoperative value of MCS projected for patients with a WOMAC Pain score of 50, b_1_ is the mean change of MCS at follow-up (again for a post-operative WOMAC Pain score of 50), b_2_ is the increment of MCS for 10 additional points of the WOMAC Pain score at baseline (i.e., the slope), and b_3_ is the change in this slope at follow-up. If the relationship between the pain score and the mental health score remained unchanged at follow-up, the coefficients b_1_ and b_3_ would be null.

We also estimated intraclass correlation coefficients from the mixed linear models, as ratios of random intercept variance to the sum of random intercept variance and residual variance.

Analyses were performed using SPSS version 22 and Stata version 13.

## Results

**Fig 1:**
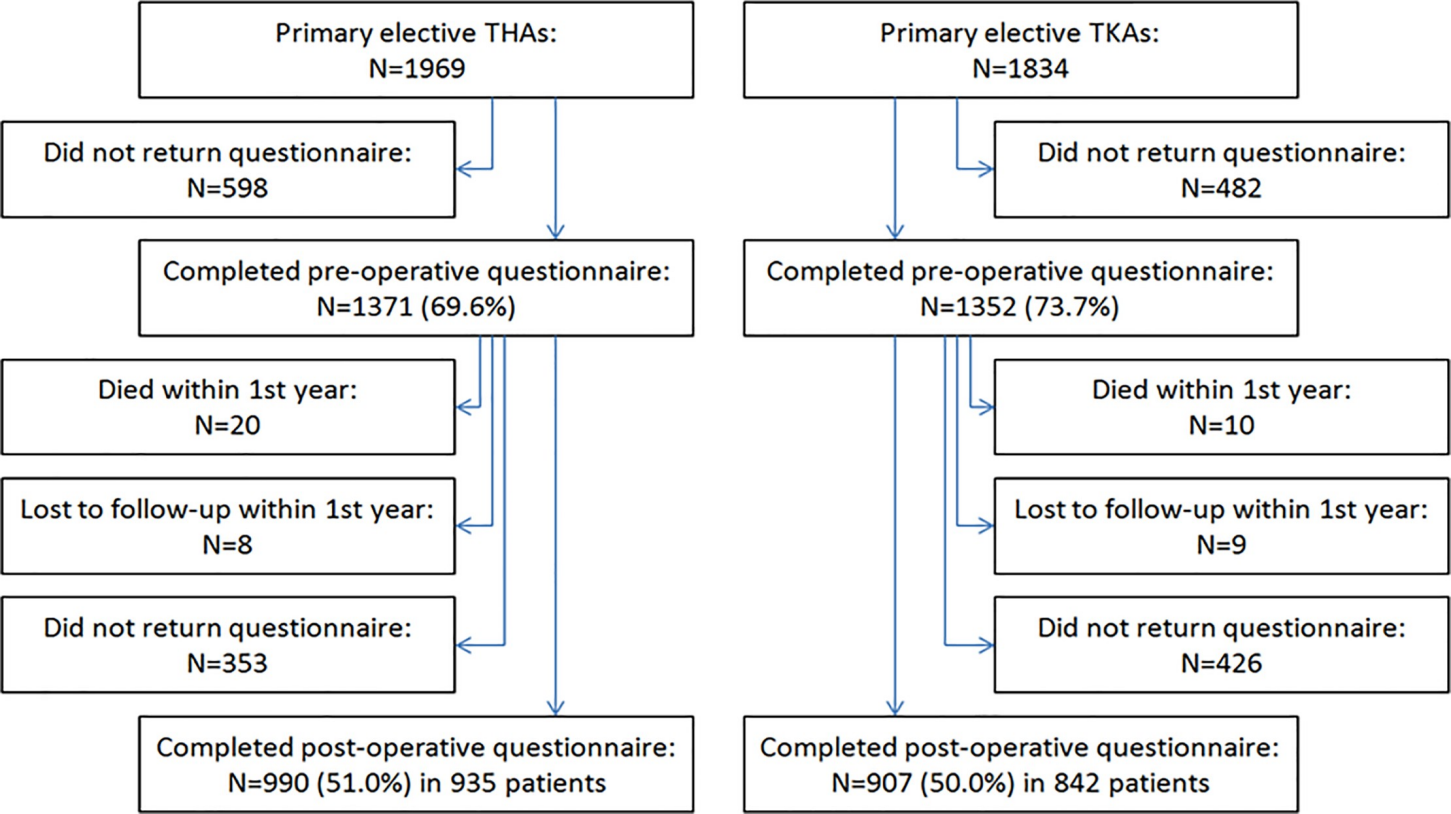
Flow-chart of the study.

During the study period, 1969 THAs and 1834 TKAs were performed at the hospital (Fig 1), and 990 THAs in 935 patients and 907 TKAs in 842 patients completed both questionnaires and were included in this analysis.

The differences between arthroplasties that were included in the analysis and those which were excluded due to unavailable questionnaire data were generally small, with few exceptions. In THA patients (we use the term somewhat loosely here, as the unit of observation is the THA, even when two THAs were performed in the same patient), the non-participants were on average younger (67.0 years vs 68.6, p = 0.009), more likely to smoke (24.5% vs 17.9%, p<0.001) and more likely to have an ASA score of 3 (21.0% vs 15.9%, p = 0.004); differences were small and statistically non-significant in terms of sex, body mass index, and prevalence of heart disease, diabetes, and high blood pressure. In TKA patients, the non-participants were also younger (70.5 years vs 71.5, p = 0.025), had a higher body mass index (30.3 vs 29.1, p = 0.018), were more likely to be women (69.8% vs 65.5%, p = 0.048), to smoke (14.4% vs 10.7%, p = 0.018) and to have an ASA score of 3 (24.4% vs 17.3%, p<0.001); differences were small and non-significant as to comorbid conditions.

Among study participants, TKA patients were older than THA patients (mean 71.5 years (SD 9.4) vs 68.6 (11.9)), they were also more likely to be female, obese, non-smokers, and more likely to suffer from diabetes or high blood pressure (Table 1). Pre-operative pain and activity levels were similar in the THA and TKA cohorts.

**Table 1:** Baseline characteristics.

	Total hip arthroplasty N = 990	Total knee arthroplasty N = 907
Women, N (%)	532 (53.7)	594 (65.5)
Age, years, mean (SD)	68.6 (11.9)	71.5 (9.4)
Age groups, N (%)		
22–59 years	214 (21.6)	96 (10.6)
60–69 years	264 (26.7)	268 (29.5)
70–79 years	332 (33.5)	352 (38.8)
80–95 years	180 (18.2)	191 (21.1)
Body mass index, mean (SD)	26.9 (4.8)	29.7 (5.3)
Obese, N (%)	232 (23.4)	398 (43.9)
Current smoker, N (%)	169 (17.1)	97 (10.7)
Current activity level (1–10), mean (SD)	3.6 (1.7)	3.6 (1.6)
Current hip/knee pain level (0–10), mean (SD)	6.1 (1.9)	6.2 (1.9)
Heart disease, N (%)	66 (6.7)	48 (5.3)
Diabetes mellitus, N (%)	99 (10.0)	151 (16.6)
High blood pressure, N (%)	499 (50.4)	568 (62.6)
ASA score at operation, N (%)		
1	109 (11.0)	42 (4.6)
2	724 (73.1)	708 (78.1)
3	157 (15.9)	157 (17.3)

Baseline characteristics of patients who underwent primary elective total hip arthroplasty or total knee arthroplasty, and were assessed one year after surgery, Geneva Arthroplasty Registry, Geneva, Switzerland, 2010–2016. ASA: American Society of Anesthesiologists

### Impact of joint replacement surgery

Perceptions of mental and physical health both improved following joint replacement. Before surgery, mean MCS scores were around 45 in both patient groups, and increased by 2–3 points at one-year follow-up (Table 2). The PCS scores started lower, at about 34, and increased by more than 10 points in THA patients, somewhat less in TKA patients. Both WOMAC scores increased sharply in THA patients (by about 2 standard deviations, from about 40 to 80), and again somewhat less in TKA patients. All improvements over time were statistically significant (all p<0.001).

**Table 2:** Patient-reported outcome scores before and after total joint arthroplasty.

	Time	Total hip arthroplasty	Total knee arthroplasty
SF12 Mental Component score	Pre-operative	44.6 (11.1)	45.2 (11.4)
	At one year	47.6 (10.5)	47.0 (11.3)
SF12 Physical Component score	Pre-operative	33.7 (7.6)	34.7 (7.4)
	At one year	44.2 (9.8)	40.8 (9.2)
WOMAC Pain score	Pre-operative	39.8 (18.1)	39.2 (17.4)
	At one year	84.4 (20.0)	73.4 (22.5)
WOMAC Function score	Pre-operative	40.6 (18.9)	43.6 (19.2)
	At one year	78.7 (21.7)	70.1 (22.7)

Mean values (SD) of global health perceptions and of symptom scores among patients who underwent primary elective total joint arthroplasty, pre-operatively and at one-year post-operative follow-up, Geneva Arthroplasty Registry, Geneva, Switzerland, 2010–2016. SF12: Short-Form 12-item questionnaire. WOMAC: Western Ontario and McMaster Universities Osteoarthritis Index 12-item questionnaire.

### Associations between symptoms and health

**Fig 2:**
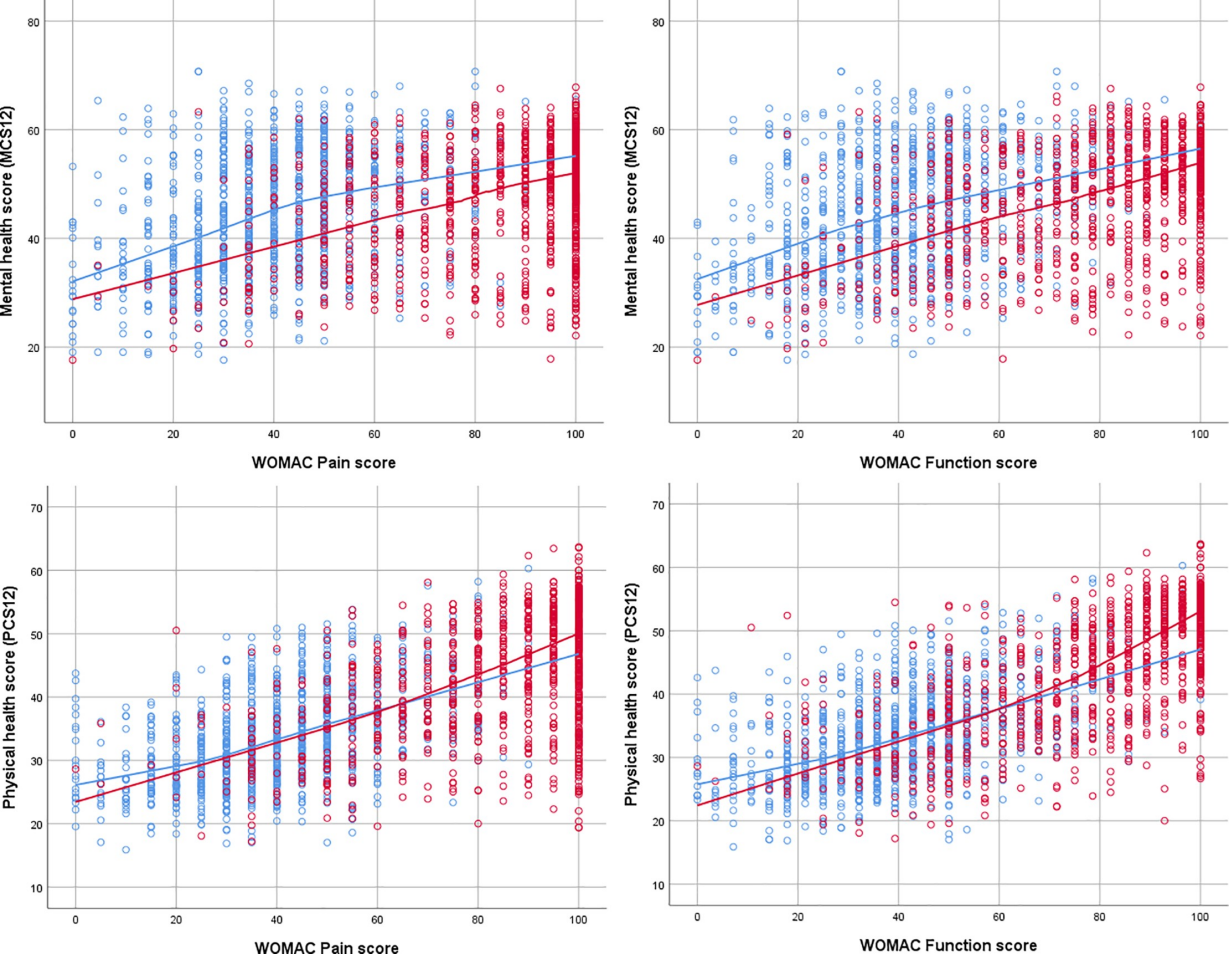
Scatter-plots of the SF12 Mental Component Score (left) and Physical Component Score (right) as a function of the WOMAC Pain score (top) and WOMAC Function score (bottom), in patients with total hip arthroplasty, before surgery (blue circles) and one year after surgery red circles). Non-parametric regression lines are superimposed (before surgery: blue line, one year after surgery: red line).

**Fig 3:**
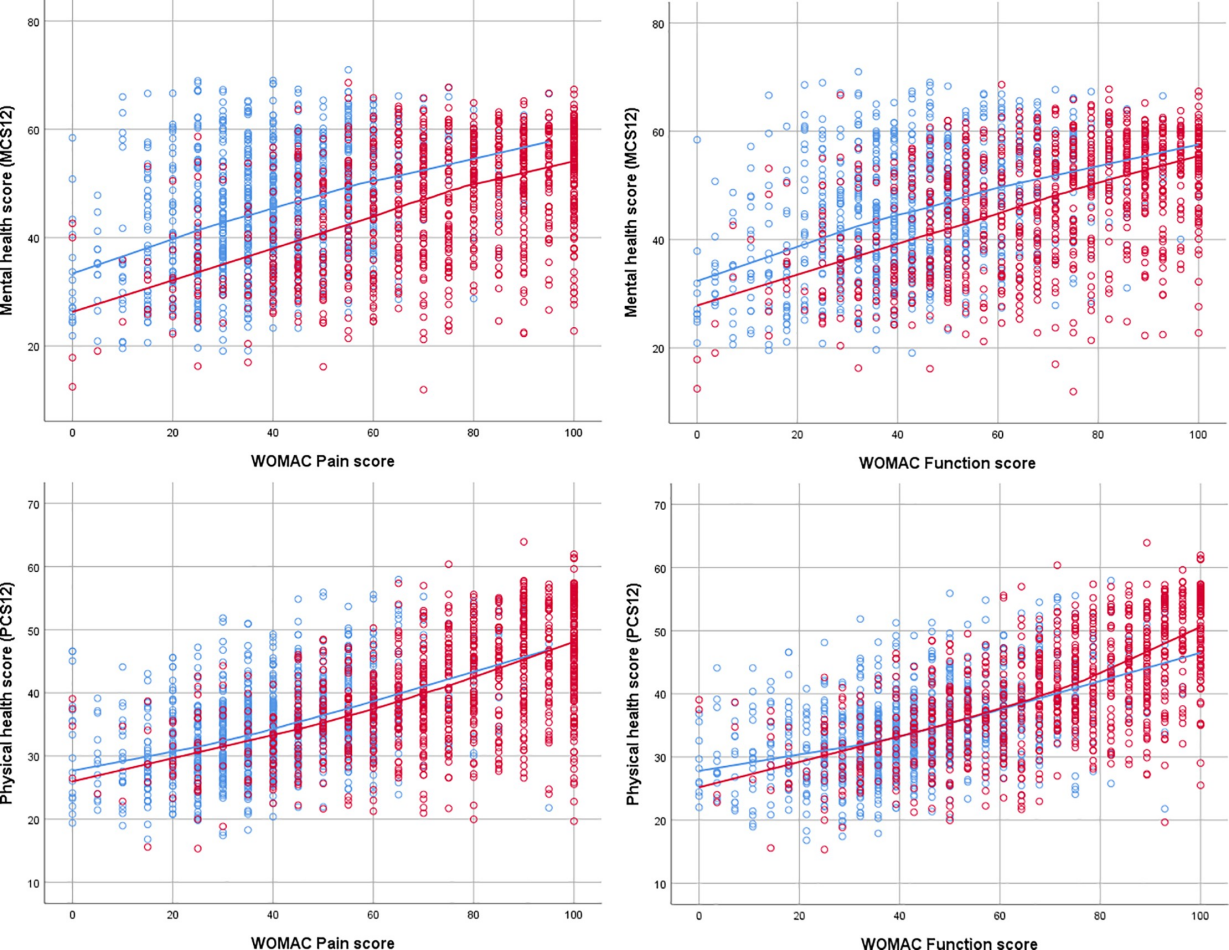
Scatter-plots of the SF12 Mental Component Score (left) and the SF12 Physical Component Score (right) as a function of the WOMAC Pain score (top) and WOMAC Function score (bottom), in patients with total knee arthroplasty (right), before surgery (blue circles) and one year after surgery (red circles). Non-parametric regression lines are superimposed (before surgery: blue line, one year after surgery: red line).

Non-parametric regression analyses showed positive associations between the WOMAC Pain and Function scores and the MCS at both points in time, in patients with THA (Fig 2 left) and TKA (Fig 3 left). The shapes of the non-parametric regression curves indicated that a linear fit was reasonable. While the two regression curves were roughly parallel, the post-operative regression curve was situated several points below the preoperative regression curve; the post-operative deficit in MCS applied across the whole spectrum of WOMAC scores. The finding was similar for the THA and TKA patients. Thus while MCS scores increased in absolute terms after surgery (Table 2), they decreased when adjusted for concurrent levels of pain or function (Table 3).

**Table 3:** Linear regression models.

		Total hip arthroplasty			Total knee arthroplasty		
Independent variable ⧩		Value at WOMAC score of 50 (intercept)	Difference for 10 points of WOMAC score (slope)	Variance explained (R^2^)	Value at WOMAC score of 50 (intercept)	Difference for 10 points of WOMAC score (slope)	Variance explained (R^2^)
	Dependent variable ➢	SF12 Mental Component Score			SF12 Mental Component Score		
WOMAC Pain score	Preoperative	46.9 (46.2–47.6)	2.3 (2.0–2.7)	0.14	47.8 (46.9–48.6)	2.4 (2.0–2.8)	0.13
	At 1 year	40.9 (39.7–42.1)	2.0 (1.7–2.3)	0.14	41.2 (40.3–42.2)	2.5 (2.2–2.8)	0.24
WOMAC Function score	Preoperative	46.6 (45.9–47.4)	2.2 (1.9–2.6)	0.14	46.8 (46.1–47.5)	2.4 (2.0–2.7)	0.16
	At 1 year	41.3 (40.3–42.3)	2.2 (1.9–2.5)	0.21	42.0 (41.2–42.9)	2.5 (2.2–2.8)	0.24
	Dependent variable ➢	SF12 Physical Component Score			SF12 Physical Component Score		
WOMAC Pain score	Preoperative	35.8 (35.3–36.2)	2.1 (1.8–2.3)	0.24	36.7 (36.2–37.2)	1.9 (1.6–2.1)	0.19
	At 1 year	35.5 (34.4–36.5)	2.6 (2.3–2.8)	0.27	35.5 (34.8–36.3)	2.2 (2.0–2.5)	0.30
WOMAC Function score	Preoperative	35.6 (35.1–36.1)	2.1 (1.9–2.3)	0.27	35.8 (35.3–36.2)	1.8 (1.6–2.0)	0.22
	At 1 year	35.5 (34.7–36.2)	3.0 (2.8–3.3)	0.46	35.6 (35.0–36.2)	2.6 (2.4–2.8)	0.41

Stratified simple linear regression models, at both points in time, with the SF12 Physical Component Score or the SF12 Mental Component Score as dependent variables, and the WOMAC Pain or Function scores as independent variables, in patients with arthroplasty of the hip or knee. 95% confidence intervals are in parentheses. SF12: Short-Form 12-item questionnaire. WOMAC: Western Ontario and McMaster Universities Osteoarthritis Index 12-item questionnaire.

For the PCS plotted as a function of the WOMAC Pain and Function scores in patients with THA (Fig 2, right) and TKA (Fig 3, right), the pre- and post-operative regression curves were close to each other; there was no systematic shift. The post-operative curves appeared to be slightly steeper at follow-up than pre-operatively, in both patient groups.

In stratified linear regression analyses, in both patient groups, the predicted values of the MCS at 50 points of the WOMAC scores were about 5–6 points lower at follow-up than pre-operatively (Table 3, top half). The slopes, i.e., increases in MCS for 10 points of the WOMAC score, remained unchanged. The proportion of variance in MCS explained by the WOMAC scores was greater at follow-up than at baseline.

In contrast, the expected value of the PCS at 50 points of either WOMAC score remained stable, at 35–36 points, in both patient groups (Table 3, bottom half). However the slopes were somewhat steeper at follow-up than before surgery. The variance in PCS explained by the WOMAC scores also increased after surgery.

Mixed linear regression models allowed a direct test of the changes in intercepts and slopes over time, after adjustment for the absolute levels of the variables. These analyses confirmed that MCS scores adjusted for WOMAC Pain and Function scores decreased significantly at the one-year follow-up (Table 4, upper half). The changes in slopes were not statistically significant. The inverse pattern was observed for the PCS (Table 4, lower half): the expected values of PCS adjusted for WOMAC Pain and Function scores remained stable, but all four slopes became significantly steeper at follow-up. The patterns were similar for the two joints. The overlap of confidence intervals on the basline-follow-up differences implies that none of the differences between THA and TKA is statistically significant. The intraclass correlation coefficients of the 8 mixed linear regression models were higher for the MCS (range 0.42 to 0.49) than for the PCS (range 0.18 to 0.27).

**Table 4:** Mixed linear regression model results.

		Total hip arthroplasty		Total knee arthroplasty	
Independent variable ⧩		Value at WOMAC score of 50 (intercept)	Difference for 10 points of WOMAC score (slope)	Value at WOMAC score of 50 (intercept)	Difference for 10 points of WOMAC score (slope)
	Dependent variable ➢	SF12 Mental Component Score		SF12 Mental Component Score	
WOMAC Pain score	Preoperative	46.5 (45.8 to 47.2)	1.9 (1.5 to 2.2)	47.3 (46.5 to 48.1)	2.0 (1.7 to 2.4)
	Change at 1 year	-4.0 (-5.3 to -2.8)	-0.4 (-0.7 to 0.0)	-5.0 (-6.0 to -3.9)	-0.0 (-0.4 to 0.4)
	P-value	<0.001	0.074	<0.001	0.89
WOMAC Function score	Preoperative	46.3 (45.7 to 47.0)	1.9 (1.6 to 2.2)	46.5 (45.8 to 47.2)	2.1 (1.8 to 2.5)
	Change at 1 year	-3.5 (-4.5 to -2.4)	-0.2 (-0.6 to 0.2)	-3.8 (-4.7 to -2.8)	0.0 (-0.3 to 0.4)
	P-value	<0.001	0.29	<0.001	0.82
	Dependent variable ➢	SF12 Physical Component Score		SF12 Physical Component Score	
WOMAC Pain score	Preoperative	35.6 (35.0 to 36.1)	1.9 (1.6 to 2.2)	36.5 (36.0 to 37.1)	1.8 (1.5 to 2.0)
	Change at 1 year	0.1 (-0.9 to 1.2)	0.6 (0.2 to 0.9)	-0.9 (-1.7 to -0.1)	0.4 (0.1 to 0.7)
	P-value	0.79	0.001	0.035	0.007
WOMAC Function score	Preoperative	35.5 (35.0 to 36.0)	2.0 (1.8 to 2.2)	35.7 (35.2 to 36.1)	1.7 (1.5 to 1.9)
	Change at 1 year	0.2 (-0.6 to 1.0)	1.0 (0.7 to 1.3)	0.1 (-0.6 to 0.8)	0.8 (0.6 to 1.1)
	P-value	0.45	<0.001	0.87	<0.001

Mixed linear regression models, with a random intercept for each patient, and for a contralateral joint nested within patient (when required), of the SF12 Physical or Mental component score as dependent variables, and the WOMAC Pain or Function scores as independent variables, in patients with arthroplasty of the hip or knee. 95% confidence intervals are in parentheses. SF12: Short-Form 12-item questionnaire. WOMAC: Western Ontario and McMaster Universities Osteoarthritis Index 12-item questionnaire.

## Discussion

This prospective study showed a substantial change in the relationships between symptoms (i.e., pain and function) and patients’ perceptions of mental health following total joint arthroplasty. Mental health scores were influenced to the same extent by pain and function after surgery as before (same slopes in the linear regression model), but their absolute level was lowered after surgery at a given level of pain and function (lower intercepts). For physical health, the absolute scores at mid-range values of pain and function were unchanged after surgery (same intercepts), but the associations became slightly stronger (steeper slopes). These results suggest that relationships between PROs are context-dependent, and that one should be careful when interpreting changes in PROs over time, especially if the changes are induced by surgery.

Total joint arthroplasty delivered very substantial pain relief and functional improvement, and increased physical health scores by more than a standard deviation on average. The improvement was less impressive for mental health (2–3 points on MCS scale), corroborating previous reports [19]. Yet, after surgery, perceived mental health was substantially lower than would have been predicted from the improvements in pain and function, as if perceived mental health was recalibrated downward. The apparent reduction in mental health was of about a half standard deviation (5 points), which can be considered as clinically meaningful [20, 21]. On the other hand, the slope of the association, which represents the sensitivity of perceived mental health to the intensity of symptoms, remained of the same magnitude as before surgery, as did the variance of mental health explained by the WOMAC Pain or Function scales. Thus pain and function remained important determinants of mental health after surgery, only with the absolute level of perceived mental health shifted downward. In other words, higher levels of function and pain relief were required after surgery than before to achieve a given level of mental health.

We observed no such systematic shift for post-operative perceptions of physical health. For an average patient, the improvement in PCS was exactly of the amount that could be predicted from the cross-sectional association observed before surgery, as though change in pain and function fully explained the improvement in perceived physical health. Furthermore, the slope of the association and the proportion of explained variance in physical health increased somewhat after surgery. This may reflect a patient’s greater focus on symptoms of osteoarthritis during postoperative rehabilitation.

The relationships beween symptom scales and measures of mental and physical health changed in a similar way for patients with both types of joint replacement, THA and TKA. This suggests that these phenomena may have general validity. It would be interesting to see if other types of interventions that have a notable impact on chronic symptoms and functional limitations (such as correction of sight or hearing, asthma medication, prosthetic limbs, or restoration of coronary flow), induce similar shifts in associations between PROs.

We can propose several possible explanations for our findings. The downward shift in MCS scores may be attributed to unfulfilled expectations. Pain and functional impairment may be less tolerable to some patients after total joint arthroplasty than before, because they may have expected better results from a surgical intervention that is often understood to be of last resort [22]. Other factors, such as a residual limp after surgery, may also have a negative psychological impact on patients [23]. Another possibility is that the surgery itself, the hospitalization and the subsequent rehabilitation were emotionally taxing, and this may have negated in part the beneficial effects of improved pain and function. Furthermore it is possible that mental health is inherently more stable than physical health, as if it reflected constant personality traits [24], and therefore is less amenable to change. This notion is supported by our observation of higher intraclass correlation coefficients for the MCS than for the PCS.

Of note, recent studies have shown a similar phenomenon: the presence of severe pain and dysfunction decreased the global perception of health (measured on a visual analog scale between 0 and 100) more strongly after total hip [25] or knee [26] arthroplasty than before. These studies used the EuroQol EQ5D instrument, which does not separate global perceptions of mental and physical health, and therefore do not replicate our findings. They do demonstrate, however, that associations between symptoms and health perceptions can be context-dependent, specifically in patients with total joint arhtroplasty.

Finally, it is possible that the measurement properties of the instruments changed following surgery and that what we have observed are variants of information bias [8]. Previous studies have described a “recalibration” to lower values of PRO instruments following total knee arthroplasty [27, 28], but the phenomenon was similar for symptom scales and for health status scales. In our study, we have observed changes in associations *between* symptom scales and health perceptions, which makes information bias less likely. In another analysis of the same patient cohorts, we observed that the self-rated health item remained completely unchanged after total joint arthroplasty [10], which provides further evidence that the measurement properties of psychometric instruments vary with context.

A strong feature of our study is the inclusion of large unselected cohorts of patients, which yields precise estimates and grants real world relevance to our results. As the same patients were assessed at baseline and at follow-up, confounding by stable patient characteristics cannot explain the observed changes.

The main limitation of our study is the lack of a clear mechanistic explanation for the observed results, which cannot be inferred from observed data. Qualitative interviews with patients before and after joint replacement surgery may improve our understanding of the contextual determinants of patients’ reports, and of the mechanisms that caused the observed changes in relationships between psychometric PRO scales. Nevertheless, the consistency of the findings in two cohorts of patients indicates that the phenomena are real. Another limitation is that we were unable to include in this analysis all possible determinants of the MCS and PCS, such as psychiatric and somatic comorbidities. However, to the extent that such determinants remain stable over the 1-year period, they would not have influenced the comparison of pre-operative and post-operative associations between WOMAC scores and SF12 scores. Finally, the study participants represented about one half of all eligible patients, mostly due to failure to return the self-report questionnaires. Study participants were somewhat older but healthier than the non-participants. However, we believe that it is unlikely that this selection process would have modified the patterns of association between health status and symptom scales, which is the main theme of this analysis.

Our results may not influence clinical decisions for individual patients, but they are relevant for the measurement of PROs as indicators of the quality of care provided by surgeons or hospitals. Clinical outcomes were not influenced in the same way even by a highly effective intervention, and their relationships were context-dependent. This suggests that measurement of multiple PROs, some disease-specific and others generic, is to be preferred in order to capture the complexity of each patient’s experience. Our results also illustrate a novel manifestation of response shift, one that affects relationships between PROs.

## Conclusions

We found that perceptions of mental health were lower after total joint arthroplasty of the hip and knee than may have been expected based on symptom relief, whereas the perceptions of physical health were as predicted by symptom relief. The reasons for these phenomena should be explored, in order to facilitate the interpretation of post-intervention PRO scores.

### Ethics approval and consent to participate

Data collection in the Geneva Arthroplasty Registry, and the use of the data for research were approved by the Research Ethics Commission of canton Geneva (CER 05–017). All patients have provided written informed consent for inclusion in the Registry.

## References

[1] Bellamy N, Kirwan J, Boers M, Brooks P, Strand V, Tugwell P et al Recommendations for a core set of outcome measures for future phase III clinical trials in knee, hip, and hand osteoarthritis. Consensus development at OMERACT III. J Rheumatol 1997;24:799–802 9101522

[2] Ostendorf M, van Stel HF, Buskens E, Schrijvers AJP, Marting LN, Verbout AJ et al Patient-reported outcome in total hip replacement. A comparison of five instruments of health status. J Bone Joint Surg (Br) 2004;86B:801–8 10.1302/0301-620x.86b6.14950 15330018

[3] Ayers DC, Bozic KJ. The importance of outcome measurement in orthopaedics. Clin Orthop Relat Res. 2013;471:3409–11 10.1007/s11999-013-3224-z 23934032 PMC3792270

[4] Franklin PD, Lewallen D, Bozic K, Hallstrom B, Jiranek W, Ayers DC. Implementation of patient-reported outcome measures in U.S. total joint replacement registries: rationale, status, and plans. J Bone Joint Surg (Am) 2014:96(Suppl 1):104–9 25520425 10.2106/JBJS.N.00328 PMC4271429

[5] Kärrholm J, Lindahl H, Malchau H, Mohaddes M, Nemes S, Rogmark C et al The Swedish Hip Arthroplasty Register Annual report 2016 Gothenburg, Sweden: Publisher Ola Rolfson, 2017. ISBN 978-91-984239-1-4

[6] Ayers DC. Implementation of patient-reported outcome measures in total knee arthroplasty. J Am Acad Ortho Surg 2017;25(suppl 1): S48–S50 10.5435/JAAOS-D-16-00631 27941415

[7] Wilson IB, Cleary PD. Linking clinical variables with health-related quality of life. A conceptual model of patient outcomes. JAMA 1995;273:59–65. 7996652

[8] Sprangers MA, Schwartz CE. Integrating response shift into health-related quality of life research: a theoretical model. Soc Sci Med 1999;48:1507–15 10400253 10.1016/s0277-9536(99)00045-3

[9] Ubel PA, Peeters Y, Smith D. Abandoning the language of “response shift”: a plea for conceptual clarity in distinguishing scale recalibration from true changes in quality of life. Qual Life Res 2010;19:465–71 10.1007/s11136-010-9592-x 20112000

[10] Perneger T, Lübbeke A. The paradox of self-rated health following joint replacement surgery. Qual Life Res 2019;28:503–8 10.1007/s11136-018-2018-x 30324584

[11] Lübbeke A, Silman AJ, Barea C, Prieto-Alhambra D, Carr AJ. Mapping existing hip and knee replacement registries in Europe. Health Policy 2018;122: 548–57 10.1016/j.healthpol.2018.03.010 29598886

[12] Ware J Jr, Kosinski M, Keller SD. A 12-Item Short-Form Health Survey: construction of scales and preliminary tests of reliability and validity. Med Care 1996;34: 220–33 8628042 10.1097/00005650-199603000-00003

[13] Leplège A, Ecosse E, Verdier A, Perneger TV. The French SF-36 Health Survey: translation, cultural adaptation, and preliminary psychometric evaluation. J Clin Epidemiol 1998;51;1013–23 9817119 10.1016/s0895-4356(98)00093-6

[14] Bellamy N, Buchanan WW, Goldsmith CH, Campbell J, Stitt LW. Validation study of WOMAC: a health status instrument for measuring clinically important patient relevant outcomes to antirheumatic drug therapy in patients with osteoarthritis of the hip or knee. J Rheumatol 1988;15:1833–40 3068365

[15] Whitehouse SL, Lingard EA, Katz JN, Learmonth ID. Development and testing of a reduced WOMAC function scale. J Bone Joint Surg (Br) 2003; 85: 706–711 12892194

[16] American Society of Anesthesiologists (ASA). ASA Physical Status Classification System. 2014. https://www.asahq.org/standards-and-guidelines/asa-physical-status-classification-system (accessed Feb 20, 2019)

[17] Zahiri CA, Schmalzried TP, Szuszczewicz ES, Amstutz HC. Assessing activity in joint replacement patients. J Arthroplasty 1998;13:890–895 10.1016/s0883-5403(98)90195-4 9880181

[18] Cleveland WS. Robust locally weighted regression and smoothing scatterplots. J Am Stat Assoc 1979;74:829–36

[19] SooHoo NF, Li Z, Chenok KE, Bozic KJ. Responsiveness of patient reported outcome measures in total joint arthroplasty patients. J Arthroplasty 2015; 30:176–91 10.1016/j.arth.2014.09.026 25449591

[20] Kazis LE, Anderson JJ, Meenan RF. Effect sizes for interpreting changes in health status. Med Care 1989;27 (3 Suppl):178–89 10.1097/00005650-198903001-00015 2646488

[21] Busija L, Pausenberger E, Haines TP, Haymes S, Buchbinder R, Osborne RH. Adult measures of general health and health-related quality of life. Arthritis Care Res 2011;63:S383–S412 10.1002/acr.20541 22588759

[22] Burton KE, Wright V, Richards J. Patients’ expectations in relation to outcome of total hip replacement surgery. Ann Rheumat Dis 1979;38:471–4 10.1136/ard.38.5.471 518147 PMC1000396

[23] Palazzo C, Jourdan C, Descamps S, Nizard R, Hamadouche M, Anract P, Boisgard S, Galvin M, Ravaud P, Poireaudau S. Determinants of satisfaction 1 year after total hip arthroplasty: the role of expectations fulfilment. BMC Musculoskeletal Disord 2014;15:53 10.1186/1471-2474-15-53 PMC3936828 24564856

[24] Cullati S, Courvoisier DS, Burton-Jeangros C. Mental health trajectories and their embeddedness in work and family circumstances: a latent state-trait approach to life-course trajectories. Sociol Health Illn 2014;36:1077–94 10.1111/1467-9566.12156 25117917

[25] Nemes S, Burström K, Zethraeus N, Eneqvist T, Garellick G, Rolfson O. Assessment of the Swedish EQ-5D experience-based value sets in a total hip repolacement population. Qual Life Res 2015;24:2963–70. 10.1007/s11136-015-1020-9 26038221

[26] Pickard AS, Hung YT, Lin FJ, Lee TA. Patient experience-based value sets. Are they stable? Med Care 2017; 55:979–84 10.1097/MLR.0000000000000802 29028757

[27] Razmjou H, Yee A, Ford M, Finkelstein JA. Response shift in outcome assessment in patients undergoing total knee arthroplasty. J Bone Joint Surg Am 2006; 88:2590–5 10.2106/JBJS.F.00283 17142408

[28] Razmjou H, Schwartz CE, Yee A, Finkelstein JA. Traditional assessment of health outcome following total knee arthroplasty was confounded by response shift phenomenon. J Clin Epidemiol 2009;62:91–6 10.1016/j.jclinepi.2008.08.004 19095168

